# Commentary: Semi-Metric Topology of the Human Connectome: Sensitivity and Specificity to Autism and Major Depressive Disorder

**DOI:** 10.3389/fnins.2016.00353

**Published:** 2016-08-03

**Authors:** Tiago Simas, John Suckling

**Affiliations:** ^1^Department of Psychiatry, University of CambridgeCambridge, UK; ^2^Cambridge and Peterborough NHS Foundation TrustCambridge, UK

**Keywords:** neuroimaging, functional connectivity, transitivity, semi-metricity, connectome

Functional Magnetic Resonance Imaging (fMRI) records the blood oxygenation level dependent (BOLD) endogenous contrast, a physiological surrogate for brain activity. Experimental and analytic procedures for fMRI remained largely unchanged in the decade following discovery of BOLD contrast, detecting localized magnitude changes in response to external stimuli. Observations of persistent patterns of activation present under a wide variety of cognitive conditions, now known as the default mode network (Raichle et al., 2001), led to significant changes in data acquisition and analysis; that is, fMRI data began to be acquired in task-absent states (so-called “rest”) and the analysis proceeded by generation of the functional connectome (Bullmore and Sporns, 2009) that putatively supported the distributed exchange of information, and supplanted localized activity as the basic unit of interpretation.

The functional connectome is constructed from nodes (brain regions) connected by edges with associated strengths (edge weights) that represent functional proximity, often inter-regional synchronicity measured by Pearson's correlation of BOLD time-series. Other strengths can be estimated; for example, coherence, cross-correlation (Salvador et al., 2005) or spectral mutual information (Granger and Hatanaka, 1964; Granger and Lin, 1994; Simas et al., 2015) which may capture alternative properties of the connectome. With this approach, a large-scale functional organization of the brain has been proposed (Bota et al., 2015) and many common mental health disorders linked to the vulnerability of particular topological elements of the connectome (Crossley et al., 2014).

Through whatever means these graphical networks are generated, complex network analysis can be applied to characterize the topography and thus the presumed flow or exchange of information that the network represents (Watts and Strogatz, 1998; Barabási and Albert, 1999; Barrat et al., 2008). Examples are replete in natural and man-made systems: computer networks, transport infrastructure, social and ecological relationships, and microstructures of the central nervous system. Up to now, complex analysis of the functional connectome has been dominated by characterization with parameters derived from a graphical network that is sparse and frequently binary (Cao et al., 2014). These networks are mostly simply created by a threshold on the edge weights, focusing then on the clique of edges with high values. Properties like small-worldness (Watts and Strogatz, 1998; Achard et al., 2006; Bassett and Bullmore, 2006; Simas, 2012; Suckling et al., 2015) can be estimated, implicitly assuming that information flows preferentially and most efficiently along paths with the fewest edges.

The role of “weak” edges has arguably been underrepresented in the complex analysis of the functional connectome (Suckling et al., 2015), although sociological theory has long recognized their central role in the distribution of information through friendship networks (Granovetter, 1973, 1983). Moreover, the complete transfer of information via shortest paths, i.e., the fewest edges between two nodes, is only possible if there is available a map of the connectome available to plan the most efficient routes, in the same way as a traveler has a map of the metro to efficiently navigate a city. It seems unlikely that the brain has to hand a representation of its own connectome, even more so given the connectome is time varying (Hutchison et al., 2013) (what is often measured by the functional connectome is a time average). More likely is that information is transferred across the entire, fully-connected network taking advantage of the proletariat of weak edges, with broadcast dynamics a potential strategy for dissemination. Nevertheless, the shortest path is a good starting point for a more expansive conceptualization.

Networks that are not transitive with edge weights representing proximities have homologs in the isomorphic distance space that are semi-metric (Klir and Bo, 1995; Simas and Rocha, 2015) (Figure 1). In other words, there are edges in the distance space that violate the triangle inequality when enforced by distance closure. Thus, it is possible to distinguish a metric edge from a semi-metric edge by determining whether the shortest path is the direct path between nodes, or if it is via a circuitous route (and there may be more than one) involving additional nodes. This is a common phenomenon; for example, although it might be difficult to communicate directly with someone with whom you have no direct relationship, it is possible to transfer messages through intermediaries with whom you are mutually acquainted.

**Figure 1:**
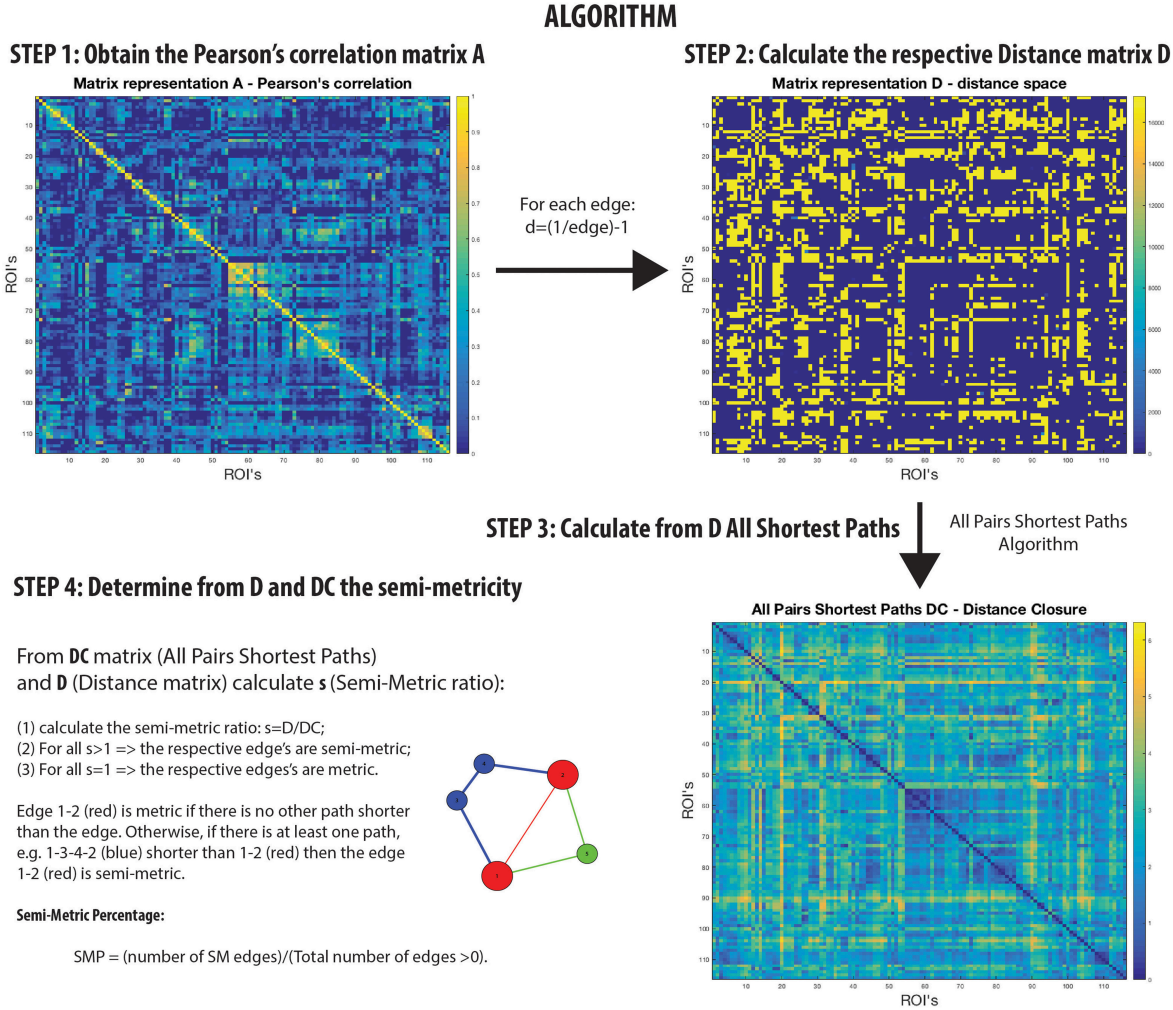
**Semi-metric analysis Algorithm on rs-fMRI with Pearson's correlation**.

A semi-metric path in the functional connectome with edge weights estimated by Pearson's correlation between regional BOLD time-series, may be interpreted as the two regions synchronously co-activating along with all other regions involved the circuitous paths (Simas et al., 2015) (Figure 1). That is, there is a dispersion of communicability across the regions. Complementarily, metric connections do not have the significant involvement of other regions, and information exchange is constrained to the two regions. All non-trivially organized networks have some degree of semi-metricity, and in the healthy human functional connectome derived with Pearson's correlation they form around 80% of all the edges (Simas et al., 2015). There is also evidence that the degree of semi-metricity (i.e., transitivity) in anatomical networks predicts functional connectivity (Goñi et al., 2014).

Semi-metric analysis of the functional connectome (Figure 1) is sensitive and specific to psychopathologies (Peeters et al., 2015; Simas and Rocha, 2015; Simas et al., 2015; Suckling et al., 2015). Both positive and negative deviations in the global proportion of semi-metric edges, relative to neurotypical individuals, have been observed in Autism and Major Depressive Disorder respectively (Simas et al., 2015), occuring consistently across individuals in similar functional connections. Psychosis was also exclusively associated with only positive changes to semi-metricity, the severity of symptoms related to the magnitude of change (Peeters et al., 2015). However in Alzheimer's disease, both directions of effect were observed, with highly idiosyncratic patterns of change (Suckling et al., 2015). Together, these studies suggest that there exists an optimum value of semi-metricity both globally and locally that is associated with healthy brain function, and that disorders have their own particular pattern of change relative to control samples.

The human brain is the most complex system known. The evidence and analytic models to measure and predict its form and function have evolved toward an understanding of brain as a network of unceasing communication. Current tomographic technologies, like fMRI, limit the detectable time resolution and we are therefore only beginning to understand the topology of the connectome and how it might form the substrate for cognition and psychopathologies. Semi-metricity, and more generally the inclusion of all the brain's connections, is a next step toward a richer description of the functional topology and, subsequently, simulation and measurement of its complex dynamics and inter-regional information transmission.

## Author contributions

Both authors made significant contributions to the drafting of the article.

## Funding

This study was funded by the UK Medial Research Council (grant: G0802226), the National Institute for Health Research (NIHR) (grant: 06/05/01) and the Behavioural and Clinical Neuroscience Institute (BCNI), University of Cambridge. The BCNI is jointly funded by the Medical Research Council and the Wellcome Trust. Additional support was received from the NIHR Cambridge Biomedical Research Centre.

### Conflict of interest statement

The authors declare that the research was conducted in the absence of any commercial or financial relationships that could be construed as a potential conflict of interest.
